# Increased Asymmetry of Trunk, Pelvis, and Hip Motion during Gait in Ambulatory Children with Spina Bifida

**DOI:** 10.3390/sym13091595

**Published:** 2021-08-31

**Authors:** Melissa A. Bent, Eva M. Ciccodicola, Susan A. Rethlefsen, Tishya A. L. Wren

**Affiliations:** 1 Children’s Hospital Los Angeles, Los Angeles, CA 90027, USA; 2 Department of Orthopedic Surgery, University of Southern California, Los Angeles, CA 90007, USA

**Keywords:** spina bifida, gait, asymmetry

## Abstract

Spina bifida (SB) is caused by incomplete neural tube closure and results in multiple impairments, including muscle weakness. The severity of muscle weakness depends on the neurologic lesion level. Though typically symmetric, there can be asymmetries in neurologic lesion level, motor strength, skeletal structures, and body composition that affect patients’ gait and function. Using body segment and joint motion obtained through 3D computerized motion analysis, we evaluated asymmetry and range of motion at the hip, pelvis, and trunk in the frontal and transverse planes during gait in 57 ambulatory children with SB and 48 typically developing controls. Asymmetry and range of hip, pelvis, and trunk motion in the frontal and transverse planes were significantly greater for patients with mid-lumbar and higher level lesions compared with those having sacral/low-lumbar level lesions and controls without disability (*p* ≤ 0.01). Crutch use decreased asymmetry of trunk rotation in mid-lumbar level patients from 10.5° to 2.6° (*p* ≤ 0.01). Patients with asymmetric involvement (sacral level on one side and L3-4 on the other) functioned similarly to sacral level patients, suggesting that they may be better categorized using their stronger side rather than their weaker side as is traditional. The information gained from this study may be useful to clinicians when assessing bracing and assistive device needs for patients with asymmetric SB involvement.

## 1. Introduction

Spina bifida (SB) occurs in 3.3 per 10,000 live births in the United States. The most severe form, myelomeningocele, results from incomplete closure of the neural tube [1]. Associated impairments typically include hydrocephalus and neurogenic bowel and/or bladder, resulting in incontinence, muscle weakness, and lack of sensation in the lower extremities [2]. There are a number of systems used to classify impairments in individuals with SB, such as lesion level assessed via X-ray, International Myelodysplasia Study Group (IMSG) level assessed via muscle strength, and functional level classified using the Hoffer ambulatory level and Dias functional level [3–8]. There can be asymmetries in neurologic lesion level and motor strength, skeletal structures, and body composition. Asymmetric motor strength does not always correspond to the sensory level of dysfunction [9].

Neurologic lesion level is usually classified based on the lowest intact spinal segment on either side, even in cases of asymmetric involvement. A patient with asymmetric lesion levels and strength profiles may function very differently than their peers with more symmetric involvement. Other sources of asymmetry also exist, which may impact gait and function. From a skeletal perspective, children with SB may have pelvic obliquity secondary to scoliosis or leg length discrepancy resulting from unilateral hip dislocation. In terms of body composition, a magnetic resonance imaging study from our institution demonstrated asymmetries and heterogeneity in fat distribution in the lower extremities in children with SB [10].

A common impairment of SB at higher neurologic lesion levels is hip abductor and extensor weakness. Hip abductor and extensor weakness causes increased transverse and frontal plane trunk and pelvic motion, increased center of mass excursion, and reduced sagittal plane motion during gait [11,12]. Hip abductor weakness has been found to increase the magnitude of pelvic rotation, pelvic obliquity, and hip abduction/adduction during gait in patients with SB. This weakness and resultant excessive motion contribute to the increased energy cost of gait and decreased self-selected gait speed when compared to same aged peers without disability [1,13]. The impact of asymmetric hip abductor and extensor weakness on pelvic and trunk motion and other aspects of gait is not known.

Gait symmetry is often a treatment goal in neurologic and post-surgical populations as persistent asymmetries have been shown to lead to impaired balance, decreased gait speed, inefficiency, and increased risk of musculoskeletal injury [14–16]. However, it has also been reported that in the non-disabled population, there are inherent asymmetries in gait, with as much as 10% asymmetry in joint moments in more than 50% of subjects in one study [17]. Forczek et al. found that the highest degree of side-to-side asymmetry during the gait cycle occurred at the ankle in the sagittal plane during the swing phase of gait in healthy men and women [18]. Other studies have found asymmetries in muscle activation, step length, stride length, and ground reaction forces [19–22]. These findings highlight the fact that gait symmetry cannot always be assumed even in uninjured, typically developing populations.

Little research has been done to examine gait symmetry in patients with SB and the impact that symmetric lesion level may have on gait [23]. The purpose of our study was to evaluate asymmetries of body segment/joint position and range of motion (ROM) at the hip, pelvis, and trunk in the frontal and transverse planes during gait in ambulatory children with SB. We hypothesized that patients with SB would have greater asymmetry in body segment/joint position and ROM than peers with typical development, especially if they had asymmetry in lesion level and muscle strength.

## 2. Materials and Methods

The data for our study were retrospectively gathered from children with SB and controls without disability who were seen for previous research studies in our motion analysis laboratory (Table 1) [24,25]. Inclusion criteria for both groups were age 8–13 years and ability to ambulate with or without assistive devices. Exclusion criteria for those with spina bifida were current use of glucocorticoid or seizure medications and chronic conditions other than spina bifida and hydrocephalus. An additional inclusion criterion for controls was participation in organized sports at least 3 times/week, and exclusion criteria for controls were injury causing loss of activity for more than 2 weeks within the past 6 months. All participants and their parents provided written assent and consent to participate in research as approved by our hospital’s Institutional Review Board.

For the participants with spina bifida, neurosegmental level was determined based on manual muscle testing following the IMSG criteria. Patients were classified as either sacral/low-lumbar level (L4 and lower) or mid-lumbar level and above (mid-lumbar+, L3-4 and higher) based on the IMSG rating of their more involved limb [8]. Asymmetry of neurologic involvement was also investigated and was defined as a difference of more than one segmental level between the left and right sides (e.g., L3-4 vs. S1).

All participants underwent instrumented motion analysis to measure dynamic three-dimensional (3D) segment and joint motions of the trunk and lower extremities in the sagittal, coronal, and transverse planes while walking at a self-selected speed in their typical footwear condition and wearing orthoses if needed. Patients walked independently without assistive devices if they were able; otherwise, they used their typical assistive devices, such as a walker or crutches. Motion analysis data were collected using an 8–10 camera motion capture system (Vicon Motion Systems Ltd., Oxford, UK). Twenty-five retro-reflective markers were placed on the participant’s lower body and trunk according to a modified Plug-in-Gait model [26]. Following a static calibration trial, participants walked along a 15 m walkway at a self-selected speed. Five to ten trials of data were recorded at 120 Hz, and at least three representative gait cycles per side were averaged for analysis. Kinematic variables calculated from the gait analysis included trunk, pelvis, and hip motion across the gait cycle. The gait cycle was defined as the time between initial foot contact to the subsequent foot contact of the same limb as determined using our motion capture software.

Asymmetry of motion was calculated as the magnitude (absolute value) of difference between the two limbs for peak hip abduction and internal rotation, ipsilateral pelvic drop (downward pelvic obliquity) and internal rotation, and trunk drop (lateral lean towards the ipsilateral side) and internal rotation over the gait cycle. Range of motion (ROM) across the gait cycle was calculated for hip, pelvis, and trunk frontal and transverse plane motion as the total excursion between the maximum and minimum values for obliquity and rotation for each joint or body segment. Since the trunk and pelvis are each a single segment (left and right are derived from the same rigid body), there was only one ROM measurement for each per plane. Each limb had a separate hip ROM, and both were included in the analysis.

Asymmetry of hip, pelvis, and trunk motion in the frontal and transverse planes was compared among the mid-lumbar+, sacral/low-lumbar, and control groups using analysis of variance (ANOVA). The ROM variables were similarly compared among the same groups using ANOVA. In the mid-lumbar+ patients, the effect of assistive device use (none, crutches, or walker) on asymmetry was also examined using ANOVA. In all cases, Bonferroni post hoc tests were used following ANOVA. All statistical analysis was performed using Stata version 14.2 (StataCorp LLC, College Station, TX, USA) with a significance level of 0.05.

## 3. Results

### 3.1. Asymmetry in Controls

Controls generally demonstrated symmetric motion, differing by an average of <3° in the frontal plane and <5° in the transverse plane for all variables studied (Table 2). Around 95% of participants had asymmetry of <7° in the frontal plane. The upper limit of asymmetry was slightly larger in the transverse plane, with 95% of participants showing asymmetry < 12°.

### 3.2. Asymmetry and ROM in Spina Bifida by Functional Level

**Figure 1.**
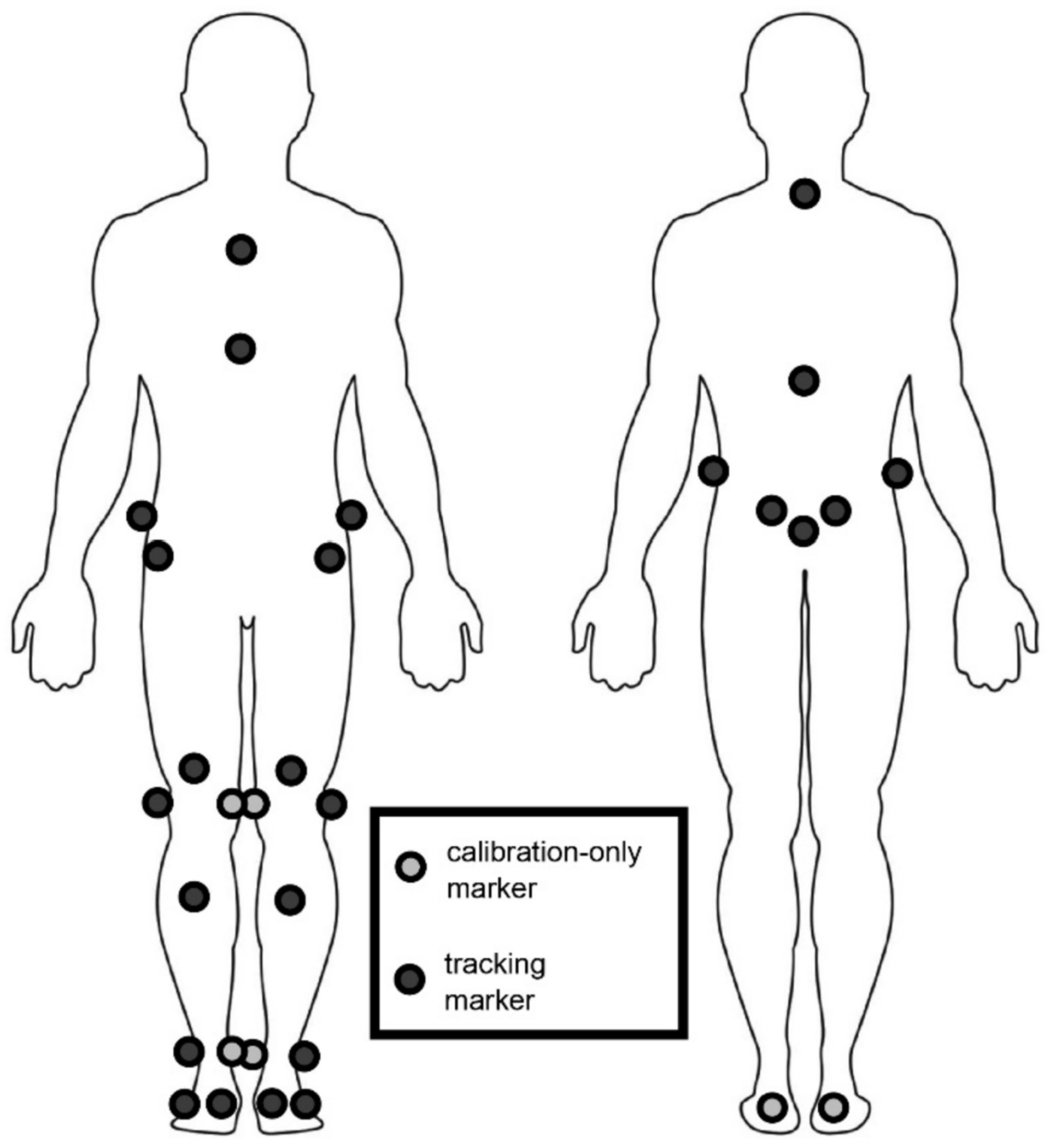
Modified Plug-in-Gait marker set.

**Figure 2.**
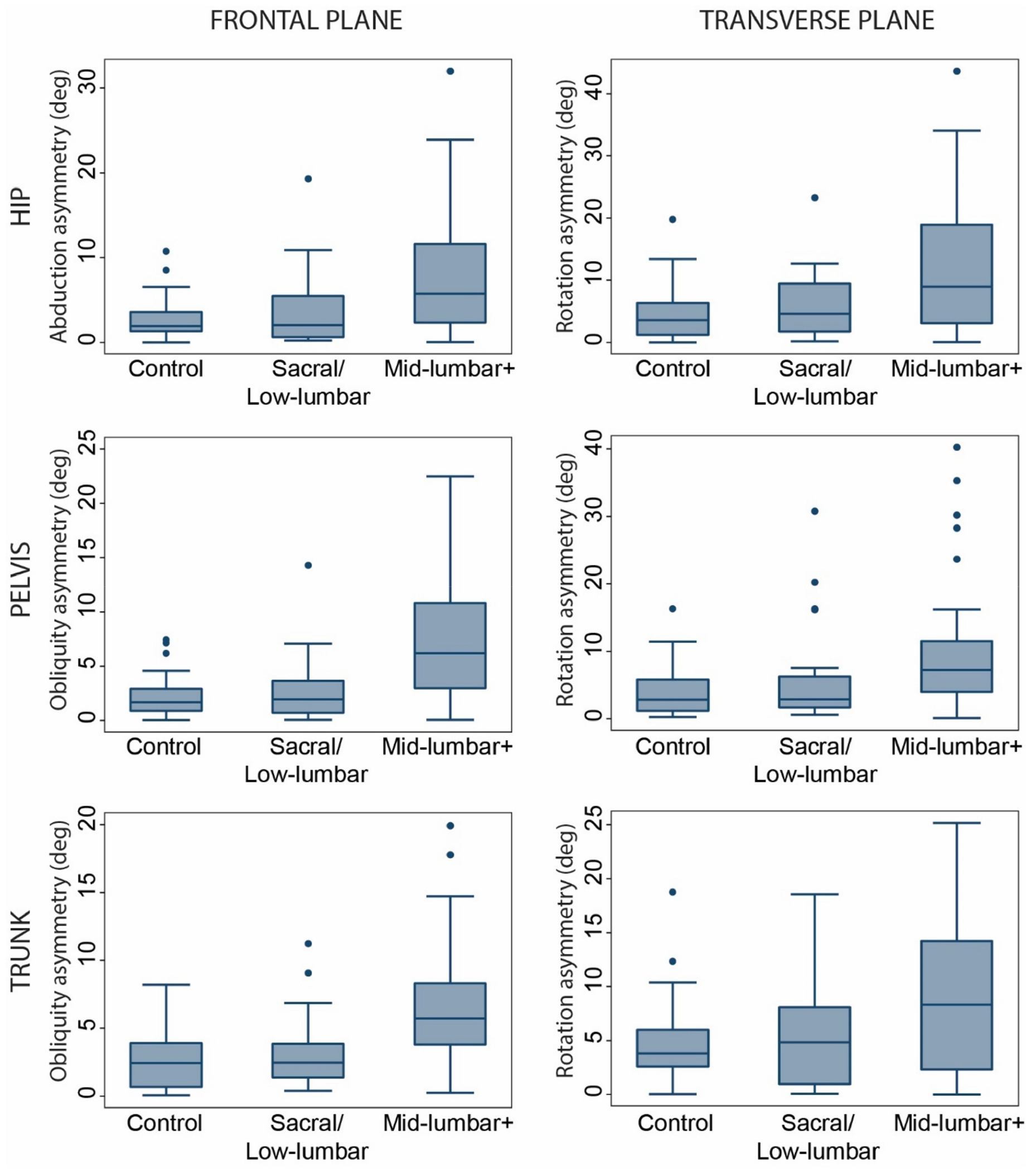
Asymmetry of gait kinematics by functional level.

Asymmetry of hip, pelvis, and trunk motion in the frontal and transverse planes was significantly greater for patients in the mid-lumbar+ group compared with the sacral/low-lumbar and control groups (Table 3 and Figure 1). Similarly, frontal and transverse plane ROM of the trunk, pelvis, and hips were much larger for the mid-lumbar+ patients compared with both the sacral/low-lumbar and control groups (Table 3 and Figure 2). There were no differences in asymmetry or ROM between the sacral/low-lumbar group and the control group. Similar results were obtained when patients with asymmetric lesion levels were excluded.

### 3.3. Effect of Assistive Devices on Gait Asymmetry

In mid-lumbar+ patients, the only difference in asymmetry based on assistive device use was lower asymmetry of trunk rotation in patients who walked with crutches. Trunk rotation asymmetry averaged 2.6° (SD 2.9) for patients with crutches compared with 10.5° (SD 6.1) for independent ambulators and 12.7° (SD 7.9) for patients using a walker (*p* ≤ 0.01). Asymmetry of the other gait parameters did not differ based on assistive device use (*p* > 0.08).

### 3.4. Patients with Asymmetric Involvement

In a small number of cases (7/57, 12%), there was asymmetric neurologic involvement. All but one of these patients had L3-4 involvement on one side and sacral level involvement or no loss on the other side. The remaining asymmetric patient had L3-4 involvement on one side and T12 involvement on the other side. This patient used a walker and showed only moderate asymmetry during gait. She had asymmetry of ≤5° for hip and pelvis frontal and transverse plane motion, 8.5° for trunk rotation, and 11.2° for trunk lateral lean.

**Figure 3.**
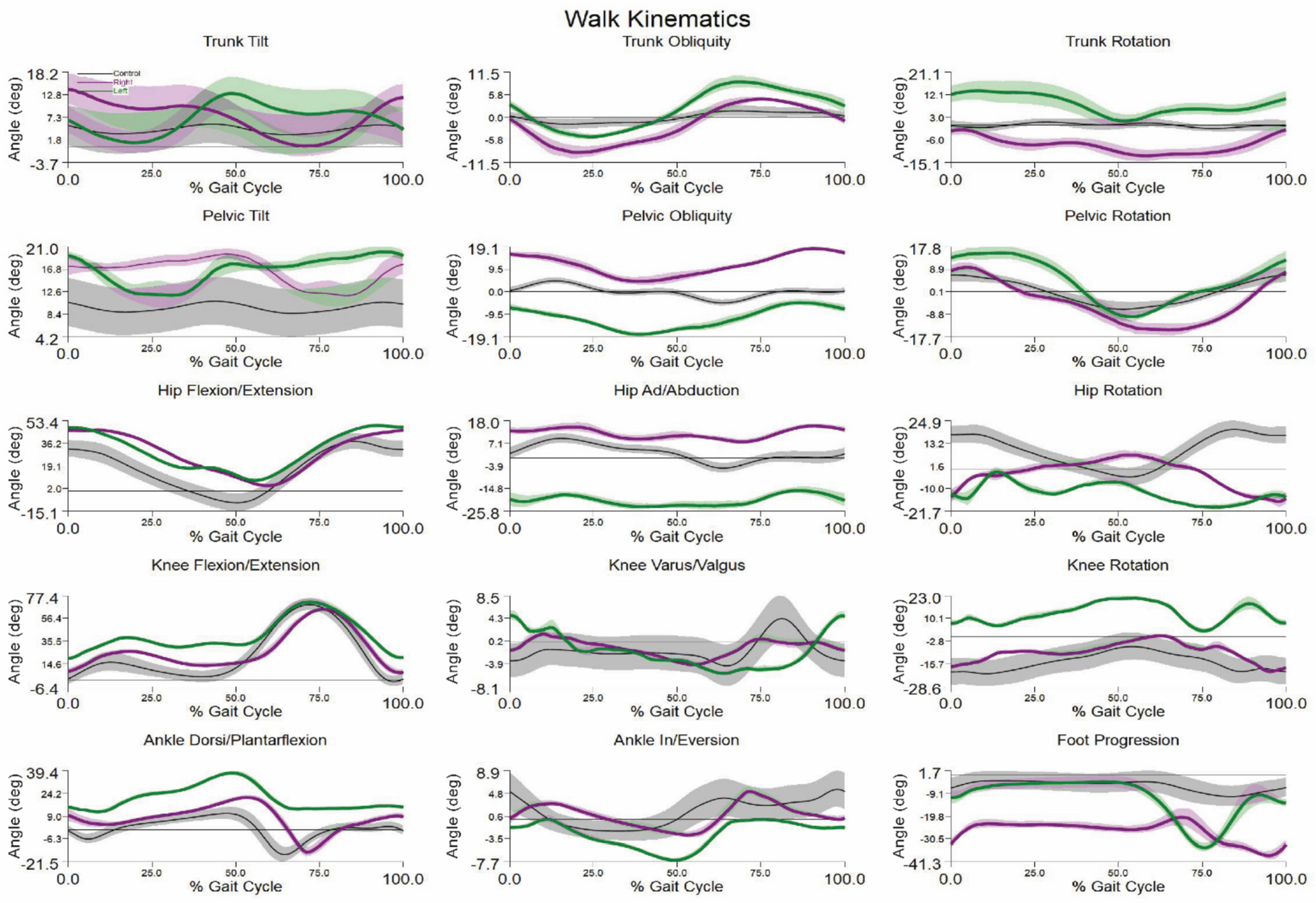
Kinematics of sample patient with asymmetric involvement showing expected gait pattern of hip abduction, pelvis and trunk drop on the weaker (left) side. Functional level is L3-4 on left and no loss on right.

Of the six patients who had L3-4 strength on one side and lower level deficits on the other side, three showed the expected pattern of greater hip abduction with compensatory pelvis and/or trunk drop on the weaker side (Figure 3). Transverse plane motion was variable with no discernable pattern being observed. The patients with asymmetric lesion levels were similar to the patients with symmetric lesion levels with the exception of greater asymmetry of trunk rotational motion in the asymmetric lesion level group (13.0° vs. 6.9°, *p* = 0.01).

## 4. Discussion

The current study is the first to assess asymmetry and ROM of hip, pelvic, and trunk kinematics in typically developing children. We found minimal hip, pelvic, and trunk asymmetry and range of motion during gait in these subjects, with 95% of subjects showing less than 7% and 12% asymmetry in the frontal and transverse planes, respectively. The finding of some asymmetry in typically developing subjects is not surprising. Forczek and Staszkiewicz examined the symmetry of gait in able-bodied women and men and found some difference between sides in lower extremity joint kinematics but no difference in temporal or phasic measures [18]. They did not, however, examine kinematics in the frontal or transverse planes which was the focus of our study.

Our SB patients with sacral/low-lumbar level lesions showed hip, pelvic, and trunk motion symmetry and ROM similar to their typically developing peers, despite the fact that some likely had weak or absent hip abductors, extensors and ankle plantarflexors. These findings are in general agreement with those of Gutierrez et al., who showed minimal gait kinematic abnormalities in SB patients with sacral level involvement and intact plantarflexors, but some kinematic deviations when the plantarflexors were absent. For purposes of statistical analysis, we grouped our sacral/low lumbar patients with and without plantarflexors together. Further study with larger numbers may allow us to separate out these groups and may better align our data with the previous literature.

Our patients with mid-lumbar+ level lesions have nearly double the hip/pelvic/trunk asymmetry and ROM that their sacral/low-lumbar level peers have. This was also seen in the study by Gutierrez et al., who showed increasing trunk and pelvic motion with increasing levels of lower extremity muscle weakness. Absence of plantarflexors, hip abductors, and extensors necessitates extra trunk and pelvic motion to maintain the center of mass over the hip joint in stance phase. Trunk and pelvic motion may also be needed to facilitate swing limb advancement in cases of insufficient plantarflexor and hamstring strength to initiate swing [11,12].

Many of our mid-lumbar+ patients used assistive devices, and this led to less trunk rotation asymmetry in crutch users. Though not specifically studied in this group, use of assistive devices, and the associated decrease in trunk rotation asymmetry, may have impacted the energy cost of walking for these patients. While it has been reported that energy expenditure was significantly less for children with SB who primarily used a wheelchair when compared to children without a disability, it is debatable if ambulatory children with SB have a significant difference in energy expenditure compared to their typically developing peers [27]. Bare et al. found that children with SB had oxygen cost and consumption more than one standard deviation above that of their age-matched peers, with increased pelvic obliquity strongly related to increased oxygen cost of walking. Their study also found that both vertical and horizontal center of mass movement throughout gait in these subjects was not significantly greater than their peers [1]. In one study that looked at ambulatory children with SB, upper and lower extremity muscle strength and peak rate of oxygen consumption were significantly lower compared to reference values [28]. Gait symmetry may play a role in energy expenditure in this population and is a possible direction for future studies [29,30].

Based on our experience, it is more common for functional neurosegmental level to be symmetric. In the current study, 12% of subjects had asymmetry of more than one neurologic level between sides. The majority of these had sacral level involvement on one side and L3-4 level involvement on the other. Though classified as mid-lumbar+ using traditional methods (based on lowest intact motor level on either side), these individuals functioned more like sacral level patients. They walked without assistive devices (no crutches or walker) and had hip, pelvic, and trunk asymmetry and ROM similar to sacral/low-lumbar level patients and those with typical development. In cases of extreme asymmetry in neurologic lesion levels between sides, it is recommended that limbs should either be classified separately, or the patients should be categorized based on the strength of the stronger side for functional purposes. Further study is needed to determine if computerized gait analysis can also be of assistance in assessing the functional implications of milder neurologic asymmetry and to determine the ability of gait analysis to predict the development of orthopedic conditions (such as hip dysplasia) over time in cases of symmetric or asymmetric lesion level in patients with SB.

The relatively small sample size was a limitation to the current study, precluding us from comparing patients with specific neurologic lesion levels, and those with other comorbidities potentially affecting gait symmetry, such as unilateral hip dislocation and scoliosis or patients with and without lesion level asymmetry. In addition, the results for patients with asymmetric lesion level were primarily descriptive because of the small sample size in this subgroup. Analysis of motion using wearables such as accelerometers or inertial measurement units could allow for assessment outside the laboratory, and inclusion of oxygen consumption data would have allowed us to directly assess the impact of excessive motion asymmetry and ROM on energy expenditure during gait. These are areas for further investigation.

In summary, patients with SB with mid-lumbar+ lesion levels exhibit greater hip, pelvic, and trunk motion asymmetry and range of motion during gait as compared to their peers with sacral/low-lumbar level lesions as well as children with typical development. Patients with asymmetric lesion levels are traditionally categorized based on the strength of the weaker limb. However, patients with one side having sacral/low-lumbar level strength function more like sacral/low-lumbar level patients, ambulating independently and exhibiting minimal hip, pelvic, and trunk asymmetry and range of motion. These patients may be better categorized according to the level of their stronger side for functional purposes as it better illustrates their ambulation and functional mobility allowing for more personalized and specific clinical care.

## Figures and Tables

**Table 1:** Participant characteristics

Variable	Control N=48	SB N=57	P-value	Asymmetric SB N=7

Sex				
Female	19 (40%)	26 (46%)	0.39	2 (29%)
Male	29 (60%)	31 (54%)		5 (71%)

Age (years)	10.9 (1.8)	11.1 (1.9)	0.71	10.6 (1.7)

Height (cm)	147.0 (13.6)	141.5 (12.8)	0.03	137.9 (8.6)

Weight (kg)	40.0 (10.9)	45.3 (18.6)	0.09	43.1 (13.6)

BMI (kg/m^2^)	18.2 (2.3)	22.0 (6.6)	0.0002	22.3 (4.8)

Functional Level	N/A			
Sacral/Low-Lumbar		22 (39%)		0
Mid-Lumbar+		35 (61%)		7 (100%)

Assistive Devices	N/A			
Sacral/Low-Lumbar				
None		21 (95%)		
Crutches		1 (5%)		
Mid-Lumbar+				
None		16 (46%)		6 (86%)
Crutches		9 (26%)		0
Walker		10 (29%)		1 (14%)

Continuous variables are reported as mean (SD) and compared using t-tests. Categorical variables are reported as n (%) and compared using Fisher’s exact test. Descriptive data are reported for the asymmetric SB subgroup. N/A = not applicable.

**Table 2.** Asymmetry of motion in typically developing children (N = 48).

	Mean	SD	95th Percentile
Frontal plane			
Hip abduction (deg)	2.6	2.2	6.6
Pelvis drop (deg)	2.1	1.7	6.2
Trunk lean (deg)	2.7	2.1	6.1
Transverse plane			
Hip rotation (deg)	4.6	4.1	11.9
Pelvis rotation (deg)	3.8	3.4	11.2
Trunk rotation (deg)	4.6	3.5	10.4

Asymmetry is expressed as the magnitude (absolute value) of difference between left and right sides in degrees.

**Table 3.** Comparison of asymmetry and ROM by functional level.

	Control N = 48	Sacral/Low-Lumbar N = 22	Mid-Lumbar+ N = 35	*p*-Value
Asymmetry				
Hip abduction (deg)	2.6 (2.2)	3.7 (4.5)	7.9 (7.0)	<0.0001 ^a,b^
Pelvis drop (deg)	2.1 (1.7)	2.8 (3.3)	7.1 (5.4)	<0.0001 ^a,b^
Trunk lean (deg)	2.7 (2.1)	3.2 (2.8)	6.3 (4.5)	<0.0001 ^a,b^
Hip rotation (deg)	4.6 (4.1)	5.8 (5.6)	11.8 (10.8)	0.0001 ^a,b^
Pelvis rotation (deg)	3.8 (3.4)	6.2 (7.7)	10.4 (10.0)	0.0004 ^a^
Trunk rotation (deg)	4.6 (3.5)	5.3 (4.7)	9.1 (7.1)	0.0005 ^a,b^
ROM				
Hip abduction (deg)	15.9 (2.9)	15.0 (4.4)	18.8 (7.4)	0.01 ^a,b^
Pelvis drop (deg)	10.1 (2.3)	9.6 (3.4)	16.1 (7.4)	<0.0001 ^a,b^
Trunk lean (deg)	5.3 (2.0)	7.7 (5.1)	16.7 (8.8)	<0.0001 ^a,b^
Hip rotation (deg)	14.6 (3.5)	19.9 (6.8)	33.4 (12.3)	<0.0001 ^a,b^
Pelvis rotation (deg)	15.2 (4.7)	19.2 (9.3)	36.1 (16.2)	<0.0001 ^a,b^
Trunk rotation (deg)	8.0 (2.2)	11.3 (8.9)	21.9 (10.3)	<0.0001 ^a,b^

All kinematic angles are reported in degrees.

a indicates *p* < 0.05 for mid-lumbar+ vs. control,

b indicates *p* < 0.05 for mid-lumbar+ vs. sacral/low-lumbar from post hoc test.

## Data Availability

Data is contained within the article.
